# The Heritability of Cognitive Aging: A Systematic Review of Longitudinal Twin Studies

**DOI:** 10.1093/geroni/igab046.3644

**Published:** 2021-12-17

**Authors:** Alice Kim, Alyssa Kam, Maxwell Kofman, Christopher Beam

**Affiliations:** 1 University of Southern California, University of Southern California, California, United States; 2 University of Southern California, Los Angeles, California, United States

## Abstract

Heritability of cognitive ability changes across late adulthood, although whether genetic variance increases or decreases in importance is not understood well. We performed a systematic review of the heritability of cognitive ability derived from longitudinal twin studies of middle-aged and older adult twins. Using the Preferred Reporting Items for Systematic Reviews and Meta-Analyses (PRISMA) guidelines, articles were identified in APA PsycINFO and Clarivate Web of Science electronic databases. Identified articles were screened by title and abstract; remaining full-text articles were then fully evaluated. Reference sections served as an additional method for identification of relevant articles. In total, 3,106 articles were identified and screened, 28 of which were included and were based on data from 10 longitudinal twin studies published from 1994-2021. There are large genetic influences on an initial level of cognitive performance across domains whereas there are small to moderate genetic influences on change in performance with age. Evidence was less definitive about whether the same or different genetic factors contribute to both level and change. Non-shared environmental influences appeared to drive individual changes in cognitive performance. Heritability tended to either be stable or decline after 65 years, possibly because of the increasing importance of non-shared environmental influences on cognitive ability. Recent studies report increases in heritability across specific subtests and domains. Shared environmental variance accounted for little variance in cognitive ability. Emerging research questions and future directions for understanding genetic and environment influences in the context of gene-environment interplay are highlighted in this review.

